# Adipokine Metabolic Drivers, Gut Dysbiosis, and the Prostate Microbiome: Novel Pathway Enrichment Analysis of the Adiposity-Based Chronic Disease—Prostate Cancer Network

**DOI:** 10.3390/cancers18020206

**Published:** 2026-01-08

**Authors:** Zachary Dovey, Elena Tomas Bort, Jeffrey I. Mechanick

**Affiliations:** 1 Department of Urology, Mount Sinai Queens and Icahn School of Medicine at Mount Sinai, 1 Gustave L. Levy Place, New York, NY 10029, USA; 2 Centre for Technology and Bioinformatics Millars, Avenue Boverot 5, 12550 Almazora, Spain; elena@ctbmillars.com; 3 Kravis Center for Clinical Cardiovascular Health at Mount Sinai Fuster Heart Hospital, Icahn School of Medicine at Mount Sinai, 1 Gustave L. Levy Place, New York, NY 10029, USA; jeffrey.mechanick@mountsinai.org

**Keywords:** adiposity-based chronic disease, prostate cancer, prostatitis, gut dysbiosis, inflammatory pathways, pathway enrichment analysis

## Abstract

Adiposity-Based Chronic Disease (ABCD) is known to increase the risk of aggressive prostate cancer (PCa), recurrent disease after treatment for localized PCa, and PCa mortality. A key mechanistic link contributing to this enhanced risk is chronic inflammation originating from excess white visceral adipose tissue (WAT; VAT) and periprostatic adipose tissue (ppWAT). Contributing to systemic inflammation is gut dysbiosis, which itself may be caused by ABCD as well as background local inflammation (prostatitis), which is common in aging men and may be exacerbated by the urinary microbiome. Investigating the molecular biology driving inflammation and its association with increased PCa risk, a recent paper applied a network and gene set enrichment to adipokine drivers in the ABCD-PCa network. It found prominent roles for MCP-1, IL-1β, and CXCL-1 in addition to confirming the importance of exposure to lipopolysaccharides and bacterial components, corroborating the role of gut dysbiosis. To further unravel the mechanistic links between ABCD and PCa risk, this critical review will discuss the current literature on prominent inflammatory signaling pathways activated in ABCD; the influence of gut dysbiosis, the urinary microbiome, and chronic prostatitis; and current hypotheses on how these domains may result in the development of aggressive PCa over a man’s life. Moreover, we performed a novel pathway enrichment analysis to further evaluate the associations between ABCD, PCa risk, gut dysbiosis, and the prostate microbiome, the results of which were partitioned into extracellular and intracellular signaling pathways. In the extracellular space, novel mechanistic links between gut dysbiosis and MCP-1, IL-1β, CXCL1, and leptin via bacterial pathogen signaling and the intestinal immune network (for IgA production), crucial for gut immune homeostasis, were found. Within the intracellular space, there were downstream signals activating chemokine and type 2 interferon pathways, focal adhesion PI3K/Akt/mTOR pathways, as well as the JAK/STAT, NF-κB, and PI3K/Akt pathways. Overall, these findings point to an emerging molecular pathway for PCa oncogenesis influenced by ABCD, gut dysbiosis, and inflammation, and further research, possibly with lifestyle program-based clinical trials, may discover novel biomarker panels and molecular targeted therapies for the prevention and treatment of PCa.

## Simple Summary

Excess Adiposity and Adiposity-Based Chronic Disease (ABCD) is a worldwide epidemic that has been linked to several cancers, including prostate cancer. One of the principal drivers is thought to be the development of chronic inflammation from visceral white adipose tissue, but a recent paper from our group that focused on prostate cancer and used network topology and gene set enrichment analysis of relevant metabolic drivers highlighted an additional source of inflammation relating to dietary exposure to lipopolysaccharides and bacterial exposure from gut and prostatic bacteria. This narrative review investigates the interplay between inflammatory signaling and exposure to gut and prostate bacteria and performs a novel pathway enrichment analysis on prominent inflammatory pathways to further unravel how excess adiposity can increase prostate cancer risk over a man’s life. Our findings support a novel molecular pathway for the development of prostate cancer linking excess adiposity, gut dysbiosis, and both systemic and local inflammation.

## 1. Introduction

Adiposity-Based Chronic Disease (ABCD) is a novel healthcare model that focuses on the prevention of overweight/obesity complications [1,2], which include several cancers. There is a significant amount of research that links ABCD to thirteen cancers, including more aggressive forms of prostate cancer (PCa) [3], and ABCD-related inflammation is believed to be one of the principal metabolic drivers underlying this link [4]. With respect to PCa, the relationship is complex. The effect of ABCD on low-risk PCa appears to be protective with reduced incidence, which may be related to lower prostate-specific antigen (PSA) levels with larger circulating volumes and therefore lower biopsy rates, reduced testosterone levels, and screening in different ethnic populations [3]. However, several epidemiological studies have demonstrated a link between ABCD and both higher risk PCa and PCa disease progression, recurrence, and mortality [3]. A Health Profession Follow-Up Study of close to 2600 men found that every 1 lb increase in weight per year from the age of 21 to diagnosis resulted in an increased chance of high-risk PCa (HR 1.47; 95% CI 1.01–2.14) in nonsmokers [5]. Other studies have shown an increased risk of biochemical recurrence (BCR) linked to BMI after surgery or radiotherapy for localized PCa [6,7], and a World Cancer Research Fund Metanalysis demonstrated that ABCD was associated with an 8–11% increased risk of metastatic PCa and PCa-specific mortality in later life [8].

As well as human epidemiological studies demonstrating an association between ABCD and PCa, evidence from animal models that ABCD-related systemic and local inflammation stemming from visceral white adipose tissue (VAT) and periprostatic white adipose tissue (ppWAT) is an important driver of this risk is compelling. In ABCD, adipocytes become hypoxic, triggering HIF-1α signaling, adipocyte cell death, and activation of M1 (pro-inflammatory) macrophages in VAT and ppWAT [9]. Alongside the development of chronic inflammation, adipokines, chemokines, and cytokines secreted by adipocytes, adipose stromal cells, immune cells (including macrophages), and cancer cells create a gradient of bioactive compounds within VAT, ppWAT, and the prostate [3], activating inflammatory pathway signaling such as PI1K/Akt, NF-KB, and JAK/STAT and promoting proliferation, angiogenesis, and survival [3,9]. Various adipokines, cytokines, and chemokines with their associated signaling pathways have been investigated as pro-inflammatory drivers of this increased PCa risk [3]. More recently, a novel network and gene set enrichment analysis was performed to investigate interactions of these adipokines, highlighting the prominent roles of monocyte chemoattractant protein-1 (MCP-1), interleukin-1β (IL-1β), and chemokine (C-X-C motif) ligand-1 (CXCL1) in the overall ABCD-PCa oncogenic network [9]. As well as outlining the interactions of prominent biomarkers, the gene set enrichment analysis (GSEA), in examining top GO categories for the overall ABCD-PCa network, demonstrated the importance of gut dysbiosis and the prostate microbiome, with ppWAT exposure activating pathways relating to both lipopolysaccharide- and bacterial-induced inflammation [9]. This ABCD-PCa network analysis corroborates the importance of gut and prostate microbiomes in the link between ABCD and PCa risk and how they both may be altered in ABCD states [10].

The role of prostatitis (chronic, acute, and histological) as a facilitator of PCa risk is also complex. Inflammatory cells are inevitably found on prostate tissue specimens throughout life, and oxidate stress with reactive oxygen species (ROS) is hypothesized to induce epigenetic alteration and other DNA mutations [10]. These changes contribute to the development of proliferative inflammatory atrophy (PIA) within prostate luminal epithelial cells, which harbor genomic alterations consistent with the initiation of PCa oncogenesis [10]. Moreover, prostatitis, which damages the epithelial cell barrier, may create a cycle of inflammation, worsening loss of the epithelial cell barrier and exposure to inflammatory stresses, contributing to PCa risk [10]. Nevertheless, a metanalysis of 25 studies with more than 20,000 patients found that PCI was associated with an overall lower risk of PCa [11], and the REDUCE trial, assessing whether Dutasteride could reduce PCa incidence, found that men who had negative prostate biopsies with PCI had fewer aggressive PCa tumors on subsequent follow-up biopsies, albeit at only 2 years [12].

In this review, we will further evaluate the associations between ABCD, gut dysbiosis, the prostate microbiome, prostatitis, and PCa risk and then report the findings of an inflammatory pathway enrichment analysis of the previously published ABCD-PCa network [9]. Consequently, a more detailed hypothesis will be generated for clinical trials of molecular targeted therapies and lifestyle biomarker panels involving patients with ABCD and PCa.

## 2. Chronic Inflammation

### 2.1. Systemic Inflammation

One of the dominant drivers linking excess adiposity to increased PCa risk is believed to be chronic systemic inflammation [9] originating from VAT. VAT consists of 90% adipose cells, but in the stromal vascular fraction (SVF), there are also immune cells (macrophages and leucocytes), endothelial cells, and adipocyte stromal cells (ASCs), all of which may secrete proteins in ABCD states that are involved in molecular pathways contributing to higher PCa risk [3]. Adipocyte hypertrophy results in ischemia, enhanced hypoxia inducible factor-1 alpha (HIF-1 alpha) signaling, angiogenesis, and macrophage proliferation [13]. Proliferating macrophages coalesce, form crown-like structures around ischemic adipocytes, and change to pro-inflammatory M1 types (as opposed to anti-inflammatory M2 macrophages), facilitating chemotaxis of other immune cells, including leucocytes [14,15]. This cascade of events results in increased secretion of a wide array of bioactive pro-inflammatory molecules, including adipokines, chemokines, and cytokines from WAT, and its SVF facilitating chronic inflammation [3]. A broad spectrum of immune cells, including mast cells, dendritic cells, lymphocytes, neutrophils, and eosinophils, are found in WAT; however, macrophages are the most abundant in ABCD, and they fall in number with weight loss [16,17]. Adipose cell death incites macrophage recruitment through activation of Toll-like and nucleotide-binding oligomerization domain (NOD)-like receptors, as well as release of chemotactic agents such as MCP1, CXCL1, and leukotriene B4 from ASCs and adipose cells [18,19]. Within this milieu, nitric oxide synthetase positive M1 type pro-inflammatory macrophages secrete pro-inflammatory cytokines (IL-1, IL-6, and tumor necrosis factor [TNF]α) and reactive oxygen species (ROS) and reactive nitrogen species, which are activated by T Helper lymphocyte cytokines such as gamma-interferon and promote both fibrosis and insulin resistance [20,21,22]. Embedded in this inflammatory network are three core pathways that link systemic inflammation and PCa risk (NF-κB, PI3K/Akt, and Janus kinases [JAKs]/signal transducers and activators of transcription [STATs]; JAK/STAT) [9,23]. The implication is that these pathways exert both endocrine and paracrine influences through gene expression and bioactive compounds, reinforcing chronic inflammation from and in VAT and ppWAT SVF, potentially contributing to prostatic inflammation, prostate inflammatory atrophy, and oncogenesis in the prostatic stroma and epithelium.

### 2.2. NF-kB Pathway

The NF-κB family of transcription factors consists of five structurally similar compounds, which are normally sequestered in the cytoplasm with inhibitory proteins. Once activated, these compounds bind to specific DNA κB enhancer regions and promote transcription of inflammatory-related target genes [24]. The NF-κB canonical and non-canonical pathways control several bioactive molecules and influence both innate and adaptive immunity [23]. The canonical pathway is activated by cytokine receptors, TNF superfamily receptors (TNFRs), T and B cell receptors, and pattern recognition receptors (PRRs) via non-infectious signals (IL-1β and TNFα), ROS, damage-associated molecular patterns (DAMPs), and pathogen-associated molecular patterns (PAMPs) [25,26]. Triggering of cell surface receptors leads to cytoplasmic degradation of an NF-κB family member by a kinase complex and nuclear translocation of other canonical members to promote downstream signaling [24]. The non-canonical pathway is activated through a separate mechanism using other members of the TNFR superfamily, with intracytoplasmic processing of an NK-κB precursor protein (p100) followed by translocation of the active non-canonical NF-κB compound to promote supplemental downstream signaling [24,27]. Central to these mechanisms is control of the differentiation and function of inflammatory T cells, which is believed to be involved in the pathogenesis of chronic inflammatory disease when the pathway signaling is dysregulated [28]. With respect to the mechanistic link between ABCD and PCa risk, activated NF-κB signaling may contribute to oncogenesis and disease progression across a number of Cancer Hallmarks [29] through transcription of related genes, including proliferation (Cyclin D1, Cyclin E, CDK2, IL-6, and Myc), survival (BEL1, BCL-2, BCL-XL, TRAF1/2, XIAP, Ciap1/2, and FLIP), angiogenesis (VEGF, IL-8, HIF1α, TNFα, CXCL1//8, 1L-1β, and IL-6), inflammation (TNFα, COX-2, INOS, MCP-1, IL-1β, IL-8, ICAM1, and ELAM1), and tumor progression and metastasis (VCAM1, ICAM1, MMP2/9, KAL1, uPA, COX2, INOS, KAL1, and ELAM1) [30].

### 2.3. PI3K/Akt Pathway

The PI3K/Akt pathway regulates several functions in the pathological inflammatory response to infection (e.g., cell survival, cell growth, and cell cycle progression), but when dysregulated, can drive oncogenesis and cancer progression; this is the most activated pathway in human cancers [31]. In normal cell function, cell membrane receptors for the PI3K/Akt pathway and associated ligands are receptor tyrosine kinases (RCKs; insulin, vascular endothelial growth factor [VEGF], fibroblast growth factor [FGF], platelet-derived growth factor [PDGF], and epidermal growth factor) and G-coupled protein receptors (GPCRs; hormones, neurotransmitters, and chemokines) [32]. Receptor binding with these ligands catalyzes the conversion of phosphatidylinositol 4,5-biphosphate (PIP2) to phosphatidylinositol 3,4,5-biphosphate (PIP3) as well as cell membrane-bound Akt activation [31]. Interestingly, PI3K/Akt signal inactivation may result from a DNA injury response via p53—phosphatase and tensin homolog (PTEN) inhibition of Akt PIP2 binding [33]. Following PI3K/Akt activation, NF-kB transcription factor and mTOR complex 1 (mTORC1) prompt cellular functions relating to survival, metabolism, and cell cycle progression [31]. In adipocytes, PI3K/Akt signaling triggered by insulin receptor (IR) binding promotes glucose uptake by membrane-bound glucose transporters (GLUTs), glucose utilization by glycogen synthase kinase, and lipogenesis by Forkhead box O subfamily (FOXO) transcription factors (e.g., FOXO1) [34]. In ABCD, elevated pro-inflammatory cytokines disrupt PI3K/Akt signaling by several mechanisms, including mTORC1 and c-Jun-N-terminal kinase (JNK) activation that blocks IR signaling by phosphorylation and expression of SOCS3 (this promotes IR ubiquitin-mediated degradation, prevents glucose uptake, and leads to insulin resistance) [32]. Moreover, without PI3K/Akt signaling, pro-inflammatory M1 macrophages may be activated by excess short-chain fatty acids (SCFAs) via Toll-like receptor 4 (TLR4); this leads to subsequent NF-κB signaling, inhibition of Tuberous Sclerosis Complex gene expression (TSC1/2), further activation of mTORC1, and eventual impairment of autophagy of damaged inflammatory cells. In short, this positive feedback loop maintains active inflammation [32].

### 2.4. JAK/STAT Pathway

The JAK/STAT signaling pathway is activated by cytokines, growth factors, and hormones and participates in metabolism, immunity, inflammation, and cellular proliferation (e.g., oncogenesis and cancer progression) [35]. When intracytoplasmic JAK is recruited and phosphorylated, STAT docking, translocation to the nucleus (as hetero- or homodimers), and DNA binding occur [35]. In ABCD, IL-6, IL-8, and leptin instigate JAK/STAT signaling [9] with IL-6-JAK/STAT pathways promoting immune escape [36]. For instance, IL-6-activated JAK/STAT signaling in a high-fat diet murine model promoted growth of myeloid-derived suppressor cells through STAT3 expression [37]. Similarly, IL-8 activated STAT3 signaling prevented apoptosis of established PCa cells in vitro [38]. Physiologically, leptin function controls appetite and energy homeostasis through hypothalamic intracytoplasmic JAK/STAT signaling [34], but several studies have demonstrated that leptin-mediated STAT3 signaling functions to influence anti-apoptosis, cellular proliferation, and migration [39]. Moreover, JAK/STAT signaling promotes lineage plasticity and cancer cell invasion/migration, which may underlie PCa treatment resistance to androgen deprivation therapy and poorer PCa outcomes [35].

### 2.5. Prostatic Inflammation and Prostate Inflammatory Atrophy

The presence of chronic inflammation as a histological finding is well established in adult prostatic tissue and has been suggested as a risk factor for PCa oncogenesis, although the association between PCI and PCa risk is complex. There is an apparent paradox: some epidemiological studies suggest PCI protects against PCa, whereas other epidemiological studies supported by molecular biology research demonstrate increased risk. For example, a metanalysis of 25 studies with more than 20,000 patients found that PCI was associated with an overall lower risk of PCa [11], and the REDUCE trial, assessing whether Dutasteride could reduce PCa incidence, found that men who had negative prostate biopsies with PCI had fewer aggressive PCa tumors on subsequent follow-up biopsies, albeit at only 2 years [12]. Despite the rationale that PCI could recruit protective immune cells, e.g., NK and CD8+ T cells that could kill emerging tumor cells [11], the epidemiological studies supporting a protective effect of PCI are likely due to collider bias [40] resulting from both negative and positive associations being confined to men who underwent prostate biopsy due to elevated PSA [40]. A key study avoiding collider bias examined inflammation in men without indication for prostate biopsy, i.e., men who were only biopsied as per protocol at the end of the Prostate Cancer Prevention Trial (PCPT) and then joined the Selenium and Vitamin E Cancer Prevention Trial (SELECT) with a repeat prostate biopsy planned later on [41]. With a mean follow-up of 5.9 years, the study found that inflammation in earlier biopsy tissue was positively associated with PCa at the later repeat biopsy [41]. The association between PCI and PCa is also supported by several other focuses of research, including the frequent finding of inflammation in the peripheral zone where most PCa arises; the proximity of PCa lesions to atrophic inflammatory lesions sharing oncogenic genetic abnormalities; animal models where PCI facilitated epithelial proliferation, cellular dysplasia, and precursor PCa lesions; and positive associations of patho-epidemiological studies linking PCI and PCa progression [40]. Although further studies to eliminate collider bias may be helpful, it seems this is a likely explanation for the observed protective effect of PCI given the weight and breadth of evidence to the contrary. Explorations of the hypothesis that PCI increases PCa risk in more detail have centered on inflammation- and ROS-related DNA damage, genomic instability, and cellular proliferation [10] as well as on oxidative stress-induced epigenetic changes with chromatin remodelers, repressor complexes, and DNA methyltransferase activity at sites of DNA damage [10,42]. Inflammatory signaling-induced oxidative stress in conjunction with androgen receptor (AR) signaling may also cause transmembrane protease serine 2 (TMPRSS2)—erythroblast transformation-specific-related gene (ERG) fusion, where TMPRS22 from chromosome 21 is translocated to ERG on the same chromosome; this fusion is known to be present in up to 50% of PCa cases and leads to ERG overexpression and cancer cell progression/survival [43].

Persistent prostatic inflammation may result in histological lesions known as “proliferative inflammatory atrophy” (PIA) that are precursors to prostate carcinogenesis and associated with prostatic intraepithelial neoplasia and PCa [43]. PIA is common in the peripheral zone of the prostate, which consists of atrophic luminal epithelial cells with a high Ki67 antigen expression, reflecting increased cellular proliferation resulting from exposure to environmental toxins and surrounding inflammatory cells [44]. PIA cells have a mixed luminal and basal epithelial histiotype, expressing cytokeratins 5 (CK5), 8 (CK8), and 18 (CK18), and, with stress, showing increased expression of hepatocyte growth factor receptor, glutathione S transferase P (GSTP1) and A1 (GSTA1), tumor antigen p53, and prostaglandin G/H synthase 2 (COX2) and decreased expression of cyclin-dependent kinase inhibitor 1B (CDKN1B) [45,46]. One study examining dissociated benign prostatic epithelial cells identified a group of luminal cells within PIA lesions with low CD38 expression (inflammation-associated luminal cells; IALs) and with increased NF-κB and reduced AR signaling, as well as increased GSTP1 and apoptosis regulator protein B cell lymphoma-2 (Bcl-2) expression [47]. These IALs had a phenotype similar to PCa cells, with a higher affinity to form colony lesions initiated by common oncogenes (e.g., AR, AKT, and MYC) [47], providing evidence of PCa precursor cells. Another single-cell RNA sequencing study on human prostate tissue identified a group of prostatic epithelial cells in areas of PIA similar in transcriptome profile and morphology to club cells in the lung [48]. These cells are enriched in surgical pathology specimens in association with localized PCa as well as PIA tissue with the TMPRSS2-ERG fusion, providing further evidence of a molecular pathway from PIA to PCa driven by inflammation [48].

### 2.6. Gut Dysbiosis

The gut microbiome is determined by the gut microbiota, which is influenced by environmental factors (e.g., dietary patterns and pollutants), and when abnormal (gut dysbiosis), can lead to PCa through the involvement of endotoxins and gut metabolites [49]. In normal conditions the commensal bacteria are primarily Firmicutes and Bacteroidetes, but also Fusobacteria, Actinobacteria, Proteobacteria, and Verrucomicrobia [50], the distribution of which may be affected by age and geography [49]. Gut dysbiosis may cause a “leaky gut” with increased permeability due to impaired expression of tight junction proteins (zonula occludens-1 [ZO-1] and occludin), causing leakage into the circulation of bacterial components such as lipopolysaccharides (LPS) and gut metabolites such as SCFAs [51]. In patients with ABCD, the ratio of Firmicutes to Bacteroidetes is reduced, Bifidobacterium levels are reduced, and fecal SCFA levels are higher. Together, these protect the gut mucosa and decrease intestinal LPS levels [52,53,54,55]. Moreover, butyrate (a 4-carbon SCFA) plays a key role in intestinal immune function and the gut mucosal barrier [53] with its anti-inflammatory and anti-neoplastic function [56]. Several studies have documented that alterations in the gut microbiome are associated with PCa development. For example, a study on 133 men who had rectal swabs at the time of prostate biopsy identified higher levels of Bacteroides and Streptococcus in those who were subsequently found to have PCa [57]. In addition, in a study on 152 men undergoing prostate biopsy, Alistipes, Rikenellaceae, and Lachonospira were specifically associated with high-risk PCa [58]. Mechanistically, altered bacterial metabolites such as lactate and SCFAs (specifically, butyrate, isobutyrate, and acetate) increase systemic IGF-1 signaling and leakage of bacterial components into the circulation (e.g., LPS and lipoteichoic acids [LTA]), contributing to systemic inflammation and altered AR signaling [51].

### 2.7. Prostate Microbiome

The urinary microbiome also affects PCa oncogenesis by causing epithelial injury that impairs the epithelial barrier. This creates a state of chronic inflammation and PIA in the prostate and exacerbates inflammatory cross talk in ppWAT [10]. Although urine is generally considered sterile, DNA sequencing has identified urinary bacterial organisms similar to those on the skin or in the gut, such as Corynebacterium, Anaerococus, Streptococcus, Staphyococcus, Lactobacillus, and Propionibacterium, as well as fungi, viruses, and protozoa [59]. The prostatic epithelium may be injured through a variety of mechanisms, including microbial, via urinary reflux, and the formation of corpora amylacea, which overcome immune defense mechanisms found in prostatic fluids [60]. In an extensive review, Sfanos et al., hypothesize that the urinary microbiome plays a role in the development of prostatic chronic inflammation and PCa oncogenesis [10]. The authors suggest that a healthy prostate with both a normal array of antimicrobial proteins (e.g., lactoferrin) and an intact epithelial barrier would not exhibit any inflammation in response to the urinary microbiome. Once the urinary microbiome is altered by infection, other forms of dysbiosis triggered by urinary tract calculi or abnormal intraprostatic urinary reflux patterns as well as dietary carcinogens, corporea amylacea, and urinary reflux may damage the epithelial barrier, activating TLR4 and causing inflammation. With the resultant generation of ROS, further epithelial cell damage and impairment of the epithelial cell barrier creates a positive feedback loop amplifying the chronic prostatic inflammation [10].

### 2.8. The ABCD-PCa Oncogenic Network

**Figure 1:**
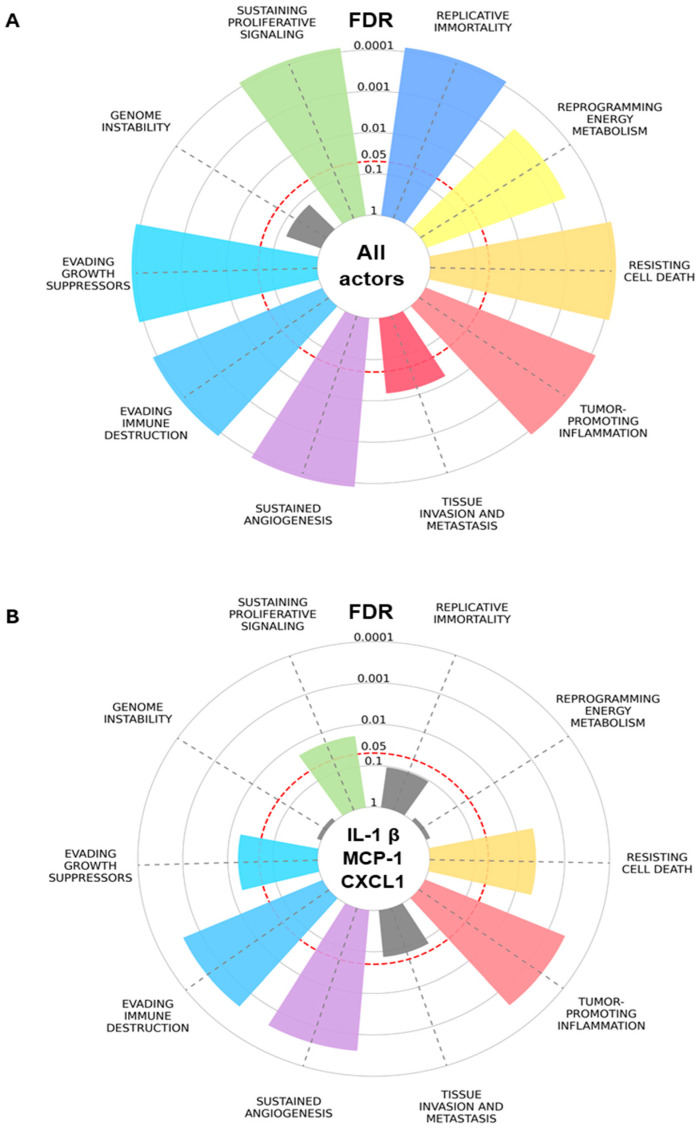
Hallmarks of Cancer enrichment plot based on ABCD-PCa biomarkers. Overrepresentation analysis for (**A**) all endo-exposome actors/biomarkers and (**B**) the top biomarkers based on betweenness centrality: IL-1β, MCP-1, and CXCL1. Significantly overrepresented Hallmarks (multiple comparison correction with false discovery rate, FDR < 0.05) are colored, while non-significant Hallmarks are represented in grey. The slice length is associated with the significance calculated for the association between obesity–prostate cancer biomarkers and the integrated Hallmark gene set. Plot generated with Cancer Hallmarks [64].

A recent 14 biomarker modular network analysis of adipokine drivers of PCa found that MCP-1, IL-1β, and CXCL1 were the most connected [9]. Gene set enrichment analysis (GSEA) of the overall network generated ranked GO categories with molecular functions relating to chronic inflammation and an immune response to bacteria and LPSs. Specifically, for MCP-1, IL-1β, and CXCL, the GSEA found GO categories pertaining to immune receptor activity, glycosaminoglycan and integrin binding, and extracellular matrix functional constituents (such as ECM remodeling), which may also relate to inflammation, the immune response to bacteria, and PCa progression within the tumor microenvironment (TME) [9]. An enrichment plot is presented that focuses on the biological effects or Cancer Hallmark functions to help unravel the mechanistic link between adipokines and PCa risk (Figure 1A,B; Table 1). The Hallmarks with the most significant enrichment for MCP-1, IL-1β, and CXCL1 are tumor-promoting inflammation, sustained angiogenesis, and evading immune destruction, whereas Hallmarks enriched in the wider network not subserved by the most prominent three are replicative immortality, reprogramming energy metabolism, and tissue invasion and metastasis (Figure 1A,B). Overlapping genes based on the Hallmarks and the ABCD-PCa network are listed according to decreasing odds ratios in Table 1.

**Figure 2:**
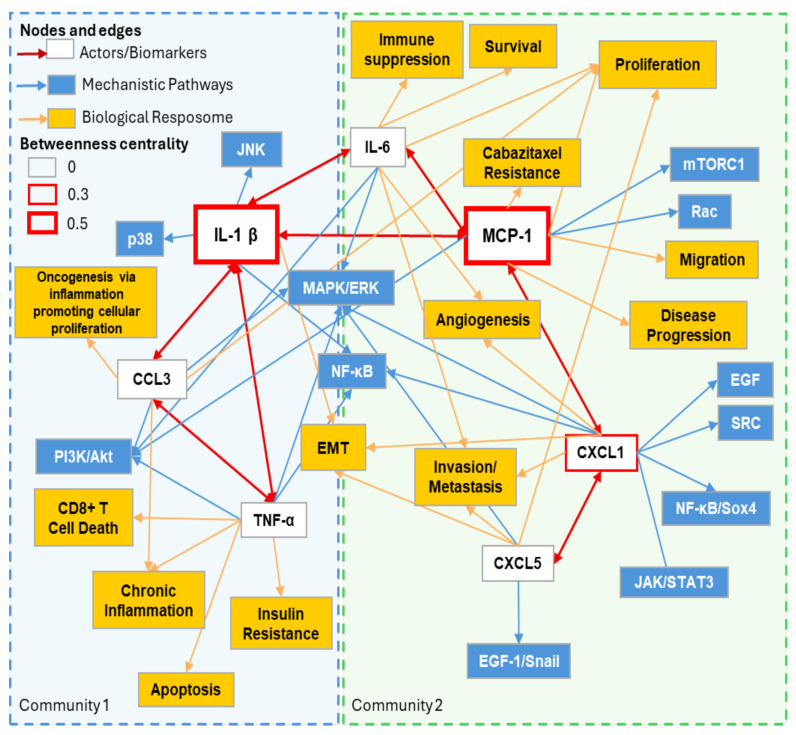
ABCD-PCa oncogenic subnetwork based on top 2 communities identified and highest centrality. Subnetwork derived from Figure 1 and Table 1. Main colored boxes (light blue and green) with dotted lines represent the top 2 communities identified by modularity calculations. IL-1β and MCP-1 are enlarged to accentuate their importance based on betweenness centrality.

A subnetwork analysis revealed two separate communities based on IL-1β–TNFα–CCL3 and MCP-1–CXCL1–CXCL5–IL-6 signaling (Figure 2). MCP-1 signaling involves significant connections to CXCL1 (itself connected to CXCL5) and IL-6, whereas IL-1β signaling involves connections with TNFα, CCL3, and IL-6. Subnetwork downstream pathways and biological effects match with the enriched Hallmarks in Figure 1B, including inflammation, angiogenesis, evading immune destruction, resisting cell death, evading growth suppressors, and cellular proliferation.

**Figure 3:**
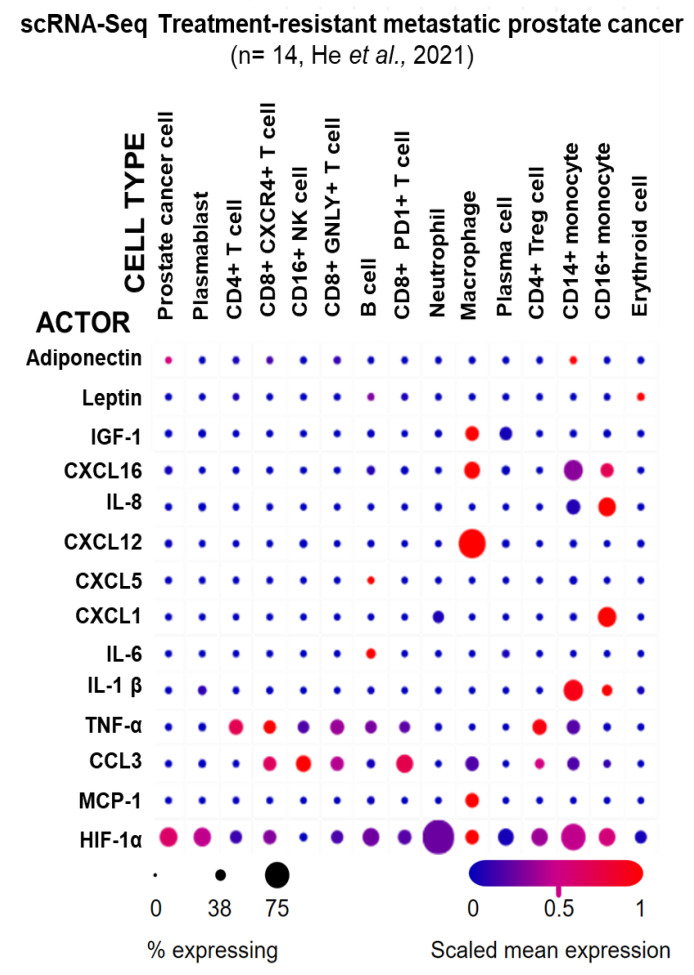
Expression of ABCD-PCa biomarkers in different cell types from treatment-resistant metastatic PCa. Single-cell RNA sequencing (scRNA-Seq) of metastatic tumors from 14 patients with treatment-resistant prostate cancer [53]. The graph was generated using the Single Cell Portal [54]. The circle size indicates the percentage of cells that express the obesity–prostate cancer endo-exposome actors/biomarker for each cell type. The color indicates the mean expression normalized to the expression of the other cell types, i.e., red indicates high expression of an actor/biomarker in a cell type relative to the other cell types [65].

To analyze biomarker cellular origin in more detail, single-cell RNA sequencing (scRNA-Seq) from metastatic treatment-resistant lethal prostate cancer from 14 patients [61] was examined via the Single Cell Portal [62] to determine which cell types in the PCa TME express the prominent biomarkers identified (Figure 3). The highest ABCD-PCa endo-exposome biomarker expression was observed in immune cells, with macrophages expressing the highest number of actors (IGF-1, CXCL16, CXCL12, CCL3, MCP-1, and HIF-1α), followed by CD14+ (inflammatory) monocytes (adiponectin, CXCL16, IL-8, IL-1β, TNF-α, CCL3, and HIF-1α) and CD16+ (innate immune system) monocytes (CXCL16, IL-8, CXCL1, IL-1β, TNF-α, and HIF-1α). Interestingly, adiponectin was only expressed in a small proportion of cancer cells, and leptin was only expressed in a small proportion of erythroid cells, precursors of red blood cells and B cells.

Leptin, adiponectin, and IGF-1 had low connectivity and did not feature highly in either of the dominant subnetworks. However, a GSEA of top ontology categories was performed (Table 2). Interestingly, leptin and IGF-1 share common molecular functions with other biomarkers that have extracellular, glycosaminoglycan, and integrin functions, suggesting a role in migration and invasion. Adiponectin is associated with DNA expression of ribosomes, chromatin, and various hormones, possibly contributing to metabolic flexibility via changes in electron transport [63]. Moreover, leptin has a strong enrichment-of-cytokine-related function, which suggests an inflammatory role. The enrichment scores of leptin and IGF-1 are higher compared to other biomarkers, and their biological effects are compatible with migration/inflammation and invasion/metastasis, respectively. This would suggest that leptin and IGF-1 function independently and are less influenced by other adipokines, whereas MCP-1, IL-1β, and CXCL1 are tightly linked in a dependency relationship, with lower individual enrichment scores. In other words, MCP-1, IL-1β, and CXCL1 are jointly expressed, whereas leptin and IGF-1 do not have an expression link with other biomarkers. Effectively, MCP-1, IL-1β, and CXCL1 seem to have co-dependent pro-tumorigenic functions in the ABCD-PCa network, whereas leptin, adiponectin, and IGF-1 function independently outside of the network communities, even though their biological effects may be similar.

**Table 1:** Cancer Hallmarks overrepresentation results based on ABCD-PCa biomarkers. This is an Overrepresentation Analysis based on the integrated Hallmark gene set and the list of identified actors/biomarkers (n = 14). Data generated with Cancer Hallmarks [64]. The top 3 actors/biomarkers based on network betweenness centrality are shown in bold in the overlapping obesity–prostate cancer biomarkers list. The odds ratio represents how much more likely a gene is to be overrepresented in a specific gene set compared to outside that gene set. An odds ratio greater than 1 indicates overrepresentation. The false discovery rate (FDR) is a significance measure that corrects for multiple comparisons (FDR < 0.05 indicated in bold).

Hallmark of Cancer	Overlapping Genes/Genes in Category	Overlapping Obesity–Prostate Cancer Endo-Exposome Actors/Biomarkers List	Odds Ratio	False Discovery Rate (FDR)
**Sustained angiogenesis**	**13**/796	IL-8, IL-6, IGF1, CXCL12, LEPTIN, MCP-1, HIF-1A, TNF-A, CXCL5, CXCL16, CCL3, **IL-1β, CXCL1**	163.15	**<0.0001**
**Evading growth suppressors**	**14**/3288	IL-8, IL-6, IGF1, CXCL12, LEPTIN, **MCP-1**, ADIPONECTIN, HIF-1A, TNF-A, CXCL5, CXCL16, CCL3, **IL-1β**, **CXCL1**	104.73	**<0.0001**
**Sustaining proliferative signaling**	**14**/3574	IL-8, IL-6, IGF1, CXCL12, LEPTIN, **MCP-1**, ADIPONECTIN, HIF-1A, TNF-A, CXCL5, CXCL16, CCL3, **IL-1β**, **CXCL1**	93.07	**<0.0001**
**Evading immune destruction**	**10**/749	IL-6, CXCL12, **MCP-1**, TNF-A, HIF-1A, CXCL5, CXCL16, CCL3, **IL-1β**, **CXCL1**	44.95	**<0.0001**
**Resisting cell death**	**12**/1941	IL-6, IGF1, CXCL12, LEPTIN, **MCP-1**, HIF-1A, TNF-A, CXCL5, CXCL16, CCL3, **IL-1β**, **CXCL1**	33.83	**<0.0001**
**Tumor-promoting inflammation**	**9**/769	IL-6, CXCL12, **MCP-1**, TNF-A, CXCL5, CXCL16, CCL3, **IL-1β**, **CXCL1**	32.31	**<0.0001**
**Replicative immortality**	**6**/547	IL-6, IGF1, CXCL12, **MCP-1,** HIF-1A, TNF-A, CXCL5	26.73	**<0.0001**
**Reprogramming energy metabolism**	**5**/740	IL-6, IGF1, LEPTIN, ADIPONECTIN, HIF-1A	11.21	**0.0005**
**Tissue invasion and metastasis**	**6**/2318	IGF1, CXCL12, **MCP-1**, TNF-A, HIF-1A, **IL-1 Β**	4.19	**0.0148**
**Genome instability**	**2**/747	TNF-A, IGF1	3.82	0.1519

**Table 2:** Top 5 GO molecular functions for actors in the ABCD-PCa oncogenic network.

Actor/Biomarker	GO ID	GO Name	Leading Edges IDs/Gene Set Size	Normalized Enrichment Score	False Discovery Rate (FDR)
	GO:0140375	Immune receptor activity	103/140	1.800	<0.0001
	GO:0005178	Integrin binding	97/157	1.720	0.0003
IL-1β	GO:0005201	Extracellular matrix structural constituent	106/161	1.647	0.0005
	GO:0005539	Glycosaminoglycan binding	132/234	1.646	0.0005
	GO:0030546	Signaling receptor activity	234/486	1.551	0.0052
	GO:0140375	Immune receptor activity	93/140	1.688	<0.0001
	GO:0003823	Antigen binding	48/65	1.627	0.0005
MCP-1	GO:0005201	Extracellular matrix structural constituent	116/162	1.600	0.0012
	GO:0005178	Integrin binding	97/157	1.584	0.0015
	GO:0005539	Glycosaminoglycan binding	132/234	1.568	0.0030
	GO:0140375	Immune receptor activity	99/140	1.654	0.0047
	GO:0003823	Antigen binding	42 /65	1.616	0.0047
CXCL1	GO:0061134	Peptidase regulator activity	90/215	1.505	0.0185
	GO:0005178	Integrin binding	96/157	1.486	0.0237
	GO:0015026	Glycosaminoglycan binding	138/234	1.461	0.0370
	GO:0140375	Immune receptor activity	87/140	1.941	<0.0001
	GO:0005178	Integrin binding	96/157	1.869	<0.0001
Leptin	GO:0050840	Extracellular matrix binding	34/55	1.859	<0.0001
	GO:0019955	Cytokine binding	92/143	1.844	<0.0001
	GO:0005126	Cytokine receptor binding	133/255	1.822	<0.0001
	GO:0003735	Structural constituent of ribosome	115/158	2.725	<0.0001
	GO:0030527	Structural constituent of chromatin	27/49	2.013	0.0018
Adiponectin	GO:0009055	Electron transfer activity	52/104	1.746	0.0479
	GO:0051427	Hormone receptor binding	7/30	1.738	0.0399
	GO:0070182	DNA polymerase binding	9/20	1.659	0.0759
	GO:0008307	Structural constituent of muscle	26/42	2.357	<0.0001
	GO:0033336	Transmembrane transporter binding	49/129	1.893	0.0033
IGF	GO:0005201	Extracellular matrix structural constituent	83/161	1.831	0.0098
	GO:0019838	Growth factor binding	56/134	1.802	0.0097
	GO:0005539	Glycosaminoglycan binding	96/234	1.796	0.0091

Gene set enrichment analysis of prostate cancer tissue data from The Cancer Genome Atlas showing enriched molecular functions with high expression levels of different biomarkers based on median expression (n = 494) using WebGestalt and the GO molecular functions database. A weighted set cover method was used to group similar significant gene sets. GO ID is the GO unique identifier for gene sets related to a molecular function. GO name is the GO molecular function gene set name. Leading edges IDs are the number of genes contributing to the enrichment score. Gene set size is the total number of genes in a GO gene set. The Normalized Enrichment Score indicates how overrepresented a gene set is in the high expression group compared to randomized gene lists. The false discovery rate (FDR) is a probability test value adjusted for multiple comparisons using the Benjamini–Hochberg method. This adjustment helps identify false positives, ensuring that the significant results are more reliable. FDR < 0.05 is significant (shown in bold).

### 2.9. Pathway Enrichment Analysis

Given the possibility that leptin, adiponectin, and IGF-1 signaling functions independently of the ABCD-PCa network and is possibly linked to gut dysbiosis and the prostate microbiome, an inflammatory pathway enrichment analysis might uncover novel mechanistic interactions. To perform this analysis, PCa RNA sequencing data from TCGA was obtained via the online cancer genomics database cBioPortal (RRID:SCR_014555) [64]. A differential expression analysis was conducted using the “Explore Selected Studies” function and compared two patient groups categorized by the median expression of the target biomarker (high vs. low expression). This analysis identified variations in gene expression levels between the groups. The resulting differential expression data was then transferred to WebGestalt (webgestalt.org) for enrichment analysis using the GSEA method. The selected functional databases included “Wikipathways Cancer” and “KEGG.” Through GSEA, the most significantly enriched pathways in PCa associated with high or low biomarker expression were identified. Finally, the results were illustrated in a diagram incorporating the false discovery rate (FDR), a statistical measure accounting for potential false positives.

**Figure 4:**
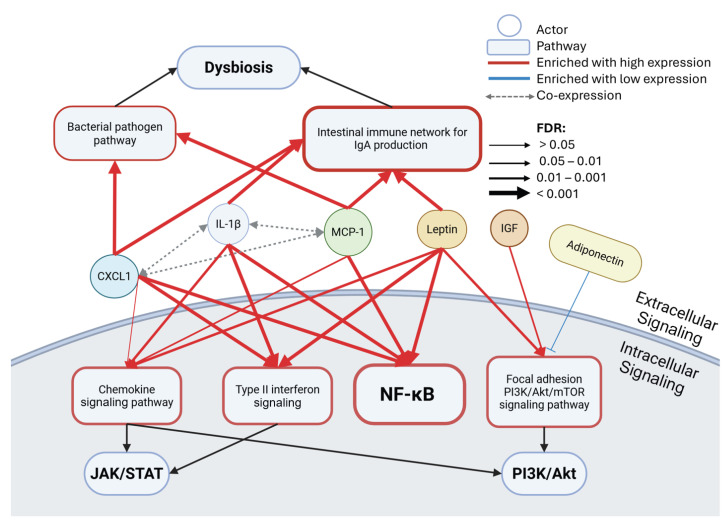
Pathway enrichment results in the ABCD-PCa oncogenic network. Gene set enrichment analysis (GSEA) of prostate cancer tissue data from The Cancer Genome Atlas (TCGA) showing enriched Wikipathways Cancer or KEGG pathways for low or high expression levels of different actors based on median expression (n = 494) using WebGestalt. Co-expression was found in the previous analysis, the ABCD-PCa modular network analysis [4]. The false discovery rate (FDR) is correlated with arrow thickness. FDR < 0.05 is significant. Black arrows indicate associated pathways, red arrows show enrichment with high expression of the actor, and blue arrows show enrichment with low expression of the actor. The thickness of the border in the pathway boxes indicates the number of connections.

After observing shared molecular functions between leptin, IGF, and the three dominant biomarkers, the common pathways were examined via a GSEA of the pathway databases Wikipathways Cancer and KEGG. The pathways involving JAK/STAT, NF-κB, PI3K/Akt signaling, and dysbiosis (Figure 4, Table 3) were found to be entrenched in several of the key biomarkers assessed in the ABCD-PCa modular network analysis [9]. There were two common pathways enriched for leptin, IL-1β, CXCL1, and MCP-1: NF-κB signaling and the intestinal immune network for IgA production. Because NF-κB may contribute to oncogenesis via the activation of the specified Cancer Hallmarks, including cell survival, the emergent link with dysregulated intestinal immunity potentially promoting immune escape further infers a PCa cellular survival mechanism.

Controlling microbiota composition and preventing excessive immune activation now appears to be an important management strategy in PCa [66]. Gut dysbiosis is disruptive, leading to immune dysregulation, increased intestinal permeability, and chronic inflammation [67]. These changes create a systemic environment that supports the development and progression of PCa, highlighting the importance of the gut–immune axis in cancer biology [67,68]. Moreover, IgA could be involved in immunosuppression, as eliminating IgA+ cells allowed cytotoxic T cell eradication of PCa cells in a mouse model [69]. Gut dysbiosis and defective IgA production is also associated with NF-κB inflammation signaling, which increases pro-inflammatory cytokine production [70].

Interestingly, MCP-1 and CXCL1 have been associated with enriched bacterial pathogen pathways [71], hinting at possible bacterial-driven inflammation, in keeping with the hypothesis of inflammation and the urinary microbiome already discussed. Type II interferon was enriched in patients with high CXCL1, IL-1β, and leptin, an upstream signaling pathway to JAK/STAT, which is also associated with an inflammatory response. As expected from the molecular function results, the chemokine signaling pathway was significantly enriched for leptin, IL-1β, and MCP-1, although non-significantly for CXCL1. Within this pathway, the genes involved in JAK/STAT and PI3K/Akt signaling are commonly found as part of its downstream signaling. IGF and leptin have also been seen to be associated with PI3K/Akt, but via the focal adhesion PI3K/Akt/mTOR signaling pathway, in line with the molecular function results that link the biomarkers with extracellular, glycosaminoglycan, and integrin functions crucial for focal adhesion signaling and migration/invasion [72]. By contrast, low levels of adiponectin have shown a trend to enrich the PI3K/Akt/mTOR pathway (FDR = 0.1), emphasizing potential anti-tumorigenic effects.

### 2.10. Implications for Prostate Cancer Management

Overall, these results corroborate the hypothesis that excess adiposity (with excess VAT and ppWAT) and gut dysbiosis (with additional influence from the urinary microbiome) may result in chemokine, NF-κB, JAK/STAT, and PI3K/Akt signaling via the highlighted biomarkers, presenting evidence for strong network-based classifier relationships between PCa, excess adiposity, and the gut microbiome in patients with ABCD. These findings also underscore the importance of lifestyle programs to reduce excess adiposity and dampen this dysbiosis–immunological axis as a prevention and treatment strategy for increased PCa risk. Several clinical studies have already examined the influence of lifestyle on PCa tumor biology in patients without necessarily focusing on the impacts of lifestyle on gut dysbiosis, inflammatory pathway signaling, or the metabolic drivers outlined above. For example, ref. [73] examined tumor and benign tissue in 402 overweight and normal weight men with PCa, 95% of whom had localized disease. Fifteen gene sets were found to be overexpressed in obese men, five of which functioned for chromatin modification and remodeling, which is linked to DNA mutation burden. Demark-Wahnefried et al. (2017) investigated weight loss in presurgical PCa patients showing increased expression of the proliferative marker Ki67 in malignant epithelium (potentially biologically disadvantageous) as well as reduced expression of genes related to insulin secretion (EFNA5) and increased expression of immune response genes (MRC1, HLA-PB1, and CD86) and DNA repair genes (biologically advantageous) [74]. A follow-up study from the same group found that weight loss was associated with loss of lean muscle and muscle catabolism, activating mitochondrial rather than glycolytic pathways. The increased Ki67 was highly linked to loss of lean muscle mass and so could potentially be reversed by combining lifestyle programs with both weight loss and resistance muscle training to maintain lean muscle [75]. The ExPeCT trial (a randomized controlled trial; “Examining Exercise, Prostate Cancer and Circulating Tumor Cells”) evaluated the effect of exercise in obese patients with advanced PCa on circulating tumor cells (CTCs). CTCs are of prognostic value in advanced PCa, and platelets have been shown to interact with CTCs in a process known as “cloaking” that facilitates immune escape from NK cells. A positive correlation was found between CTC and platelet count in non-exercise and overweight groups, which was not seen in the exercise or normal weight groups [76]. In an early landmark study, Ornish et al. investigated a lifestyle intervention involving diet, physical activity, and mindfulness-based stress reduction (MBSR) in men under active surveillance (AS) for low-risk PCa [69,70]. In the 30 recruits, there was reduced expression of 453 genes and increased expression of 48 genes all relating to tumorigenesis in biopsy tissue. After 5 years follow-up, the intervention group had increased telomerase activity with a relative increase in telomere length from baseline compared to a decrease in the control group. A more recent randomized clinical trial examining the effects of diet and exercise in 117 ABCD patients on active surveillance for low-risk PCa found that patients were able to achieve weight loss goals and demonstrated significant improvements in insulin and c-peptide as biomarkers of glucose regulation [77]. Another recent randomized clinical trial in PCa patients on active surveillance assessed a 1-year-long diet of additional fish oil capsules and high omega 3 and low omega 6 supplements and found a significantly lower Ki-67 index (a biomarker for disease progression) on follow-up prostate biopsy tissue [78]. Moreover, Dovey et al., in a systemic review of the influence of lifestyle interventions on mental health and oncological outcomes in PCa patients, found that moderate to vigorous physical activity was especially important [79]. Improved mental health was found in 7/8 studies where physical activity was included in the lifestyle program, and improved oncological outcomes were found in 11/13 studies where physical activity was included [79].

There are several clinical trials at different stages of PCa aimed at nutritional manipulation of the gut dysbiosis to restore a healthy microbiome [79]. These involve patient groups with localized or metastatic disease and standard of care management, such as the use of prebiotics (e.g., polyunsaturated fats), specific probiotics to counteract gut dysbiosis, fecal microbiota transfer to reestablish a normal gut microbiome, as well as the use of microbial-derived metabolites known to have systemic effects on PCa tumor progression [64,80]. This is an area that has promise, and similar strategies could be incorporated into a broader lifestyle program incorporating exercise aimed at reducing excess adiposity.

## 3. Limitations

This review has important limitations, including the potential for selection and publication biases related to our examination of the literature, our choice of studies to review, and the level of evidence they present. Our pathway enrichment analysis and other results are reliant on the quality of the datasets used. The results of pathway enrichment analyses may also be affected by interaction detection bias, which may be lessened by using a corrected *p* value to filter out false discovery interactions.

## 4. Conclusions

Taking a broad perspective on a man’s modifiable lifetime risk of PCa reveals some key domains over a life that may exert influence, including abnormal adiposity causing VAT, ppWAT, and gut dysbiosis, and inherent histological prostatitis, which may be associated with changes in the urinary microbiome. One of the key mechanistic links among these domains is inflammation. A prior network analysis highlighted prominent roles for MCP-1, IL-1β, and CXCL1 with leptin, adiponectin, and IGF-1 signaling. The biological effects of the wider ABCD-PCa network in relation to the Cancer Hallmarks suggests that immune cells, and specifically macrophages, are the most important source of these bioactive compounds not only in VAT and ppWAT but also in the PCa TME itself. The pathway enrichment analysis is partitioned diagrammatically into extracellular and intracellular effects. Extracellularly, there are mechanistic links between gut dysbiosis and MCP-1, IL-1β, CXCL1, and leptin via bacterial pathogen signaling and the intestinal immune network (for IgA production), which is crucial for gut immune homeostasis. Within the intracellular space, there are downstream signals activating chemokine and type 2 interferon pathways, focal adhesion PI3K/Akt/mTOR pathways, as well as the JAK/STAT, NF-κB, and PI3K/Akt pathways. Overall, these findings point to an emerging molecular pathway to PCa oncogenesis influenced by ABCD, gut dysbiosis, and inflammation, and further research, possibly with lifestyle program-based clinical trials, may discover novel biomarker panels and molecular targeted therapies for the prevention and treatment of PCa.

## Figures and Tables

**Table 3:** Pathway enrichment analysis results.

Actor/Biomarker	Pathway Database	Gene Set ID	Description	Leading Edges IDs/Gene Set Size	Normalized Enrichment Score (NES)	False Discovery Rate (FDR)
**MCP-1**	Wikipathways Cancer	WP3929	Chemokine signaling pathway	61/165	1.44	**0.04**
	Wikipathway Cancer	WP3617	Photodynamic therapy-induced NF kB survival signaling	16/35	1.61	**<0.001**
	KEGG	hsa04672	Intestinal immune network for IgA production	34/45	1.68	**<0.001**
	KEGG	hsa05150	Staphylococcus aureus infection	46/87	1.69	**<0.001**
**IL-1B**	Wikipathway Cancer	WP619	Type II interferon signaling	27/36	1.69	**<0.001**
	Wikipathway Cancer	WP3929	Chemokine signaling pathway	75/165	1.59	**0.01**
	KEGG	hsa04672	Intestinal immune network for IgA production	32/45	1.80	**<0.001**
**CXCL1**	Wikipathway Cancer	WP619	Type II interferon signaling	18/36	1.52	**0.02**
	Wikipathway Cancer	WP3929	Chemokine signaling pathway	68/165	1.44	0.08
	KEGG	hsa05150	Staphylococcus aureus infection	46/87	1.69	**<0.001**
	KEGG	hsa04672	Intestinal immune network for IgA production	34/45	1.68	**<0.001**
**Leptin**	Wikipathway Cancer	WP619	Type II interferon signaling	21/37	1.75	**<0.001**
	Wikipathway Cancer	WP3929	Chemokine signaling pathway	82/165	1.67	**0.01**
	Wikipathway Cancer	WP3932	Focal adhesion PI3K Akt mTOR signaling pathway	119/302	1.59	**0.01**
	Wikipathway Cancer	WP3617	Photodynamic therapy-induced NF kB survival signaling	18/35	1.94	**<0.001**
	KEGG	hsa04064	NF-kappa B signaling pathway	57/102	1.82	**<0.001**
	KEGG	hsa04672	Intestinal immune network for IgA production	35/45	1.88	**<0.001**
**IGF**	Wikipathway Cancer	WP3932	Focal adhesion PI3K Akt mTOR signaling pathway	98/302	1.67	**0.04**
**Adiponectin**	Wikipathway Cancer	WP3932	Focal adhesion PI3K Akt mTOR signaling pathway	87/302	−1.58	0.10

Gene Set ID is the unique identifier for gene sets related to a pathway. Leading edges IDs are the number of genes contributing to the enrichment score. Gene set size is the total number of genes in a gene set. The Normalized Enrichment Score indicates how overrepresented a gene set is in the high expression group compared to randomized gene lists. The false discovery rate (FDR) is a probability test value adjusted for multiple comparisons using the Benjamini–Hochberg method. FDR < 0.05 is significant (shown in bold).

## Data Availability

Datasets are available in the public domain.

## Abbreviations

ABCD	Adiposity-Based Chronic Disease
AMPK	adenosine monophosphate (AMP) activated protein kinase
AP-1	activator protein 1
AR	androgen receptor
ASC	adipose stromal cell
BIM	B-cell Lymphoma 2-like protein 11
CCL	“CC” motif chemokine ligand (CC motif refers to the position of two adjacent cysteine residues in the final protein)
CXCL	CXC-chemokine ligand (CXC refers to the position of two cysteine residues near the terminal of the protein, with any other amino acid representing X in the final protein)
DOCK2	dedicator of cytokinesis 2
ECM	extracellular matrix
EGF	epidermal growth factor
EMT	epithelial mesenchymal transition
ERG	erythroblast transformation-specific-related gene
ERK	extracellular signal regulated kinase
ET-1	endothelin-1
FAK	focal adhesion kinase
FGF	fibroblast growth factor
FOXO	Forkhead box subfamily 0
GSEA	gene set enrichment analysis
JAK	Janus kinase
JNK	c-Jun proto-oncogene N-terminal kinase
MAPK	mitogen-activated protein kinase
MEK	mitogen-activated protein kinase (MAPK)/extracellular signal regulated kinase (ERK) kinase
MSC	mesenchymal stromal cell
mTOR	mammalian target of rapamycin
mTORC1	mammalian target of rapamycin complex 1
NF-κB	nuclear factor-κB
PAI1	plasminogen activator inhibitor 1
PI3K	phosphoinositide 3-kinase
PKC	protein kinase C
PIA	proliferative inflammatory atrophy
PPAR	peroxisome proliferator-activated receptor
ppWAT	periprostatic white adipose tissue
RAC	Rho GTPase-activating protein
RAF	rapidly accelerated fibrosarcoma
RANKL	receptor activator of nuclear factor-κΒ ligand
RTK	receptor tyrosine kinase
SOX4	sex determining region Y-box 4
SRC	proto-oncogene tyrosine-protein kinase
STAT	signal transducer and activator of transcription
TGFβ	transforming growth factor-β
TME	tumor microenvironment
TNF	tumor necrosis factor
TMPRSS2	transmembrane protease serine 2
VAT	visceral adipose tissue
VEGF	vascular endothelial growth factor
WAT	white adipose tissue
